# Molecular Dynamics Simulation Studies of dTTP Binding and Catalysis Mediated by YhdE Dimerization

**DOI:** 10.1371/journal.pone.0134879

**Published:** 2015-08-07

**Authors:** Nan Wang, Jiahong Jiang, Xichen Li, Hongwei Tan, Jimin Zheng, Guangju Chen, Zongchao Jia

**Affiliations:** 1 College of Chemistry, Beijing Normal University, Beijing, China; 2 Department of Biomedical and Molecular Sciences, Queen's University, Kingston, Ontario, Canada; Wake Forest University, UNITED STATES

## Abstract

YhdE is a Maf-like (multicopy associated filamentation) protein that primarily acts as dTTPase to hydrolyze dTTP into dTMP and two phosphate molecules in cell metabolism pathway. Two crystal structures of YhdE have been previously determined, representing the open and closed active site conformations, respectively. Based on the structures, we have carried out molecular dynamics simulations and free energy calculations to investigate dTTP binding to and hydrolysis by YhdE. Our results suggest that YhdE closed state is structurally more compact than its open state at room temperature. YhdE open state is a favorable conformation for dTTP binding and closed state is a structurally favorable conformation for catalytic reaction. This observation is supported by the structure of YhdE homolog in complex with a nucleotide analog. Free energy calculations reveal that YhdE dimerization occurs preferentially in dTTP binding and is favorable for successive cooperative reaction. The key residues R11, R12 and K80, are found to contribute to the substrate stabilization. Further, YhdE dimerization and binding of dTTP induce the cooperative effect through a direct allosteric communication network in YhdE from the dTTP binding sites in the catalytic center to the intermolecular β-strand in YhdE dimer.

## Introduction

*Escherichia coil* YhdE is a Maf-like (multicopy associated filamentation) protein that acts as dTTPase/UTPase in the whole cell metabolism pathway, which decompose dTTP/UTP into dTMP/UMP and pyrophosphate with an unknown mechanism [1–3]. Maf protein represent a large family of conserved proteins and was found not only in bacteria, but also in archaea and eukaryotes [4]. This protein family is generally implicated in cell division regulation but with unknown specific cellular function. Recent study on Maf proteins from *B*. *subtilis* indicated that the inhibition of cell division was associated with DNA transformation and repair [2]. It also showed that dTTPase/UTPase in Maf proteins family may be involved in DNA/RNA synthesis regulation [5], however the connection between Maf dTTPase/UTPase activity and DNA/RNA synthesis or DNA transformation lacks direct evidence. As a result the physiological roles of Maf proteins are still not clear.

**Fig 1:**
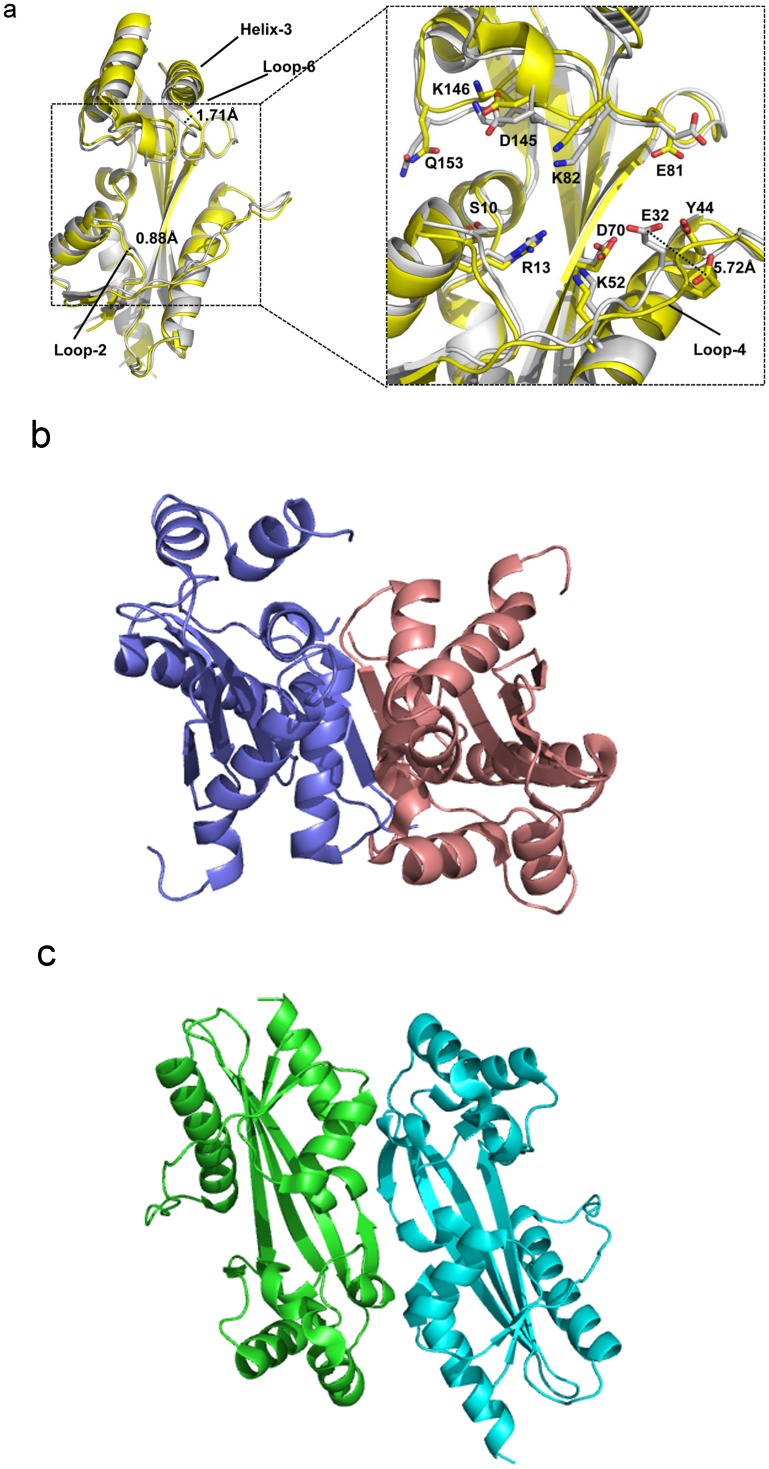
The structural comparison of YhdE-E33A in the open-state (yellow, PDB code:4P0U) and in the close state (white, PDB code:4P0E)[8]. All cartoons in this paper are prepared using PyMOL (version 0.99).

The crystal structures of several Maf proteins have been determined in both its apo form as well as bound to small solvent molecules such as phosphate or sulfate group. The structural similarity between the *M*. *jannaschii* Mj0226 dNTP pyrophosphatase (PPase) and the *E*. *coli* YjjX ITPase/XTPase [6,7], both of which are nucleotide hydrolases, provided evidences that Maf proteins appear to belong to a large protein family of nucleotide binding proteins. *E*. *coli* YhdE protein shares 46% sequence identity with Maf proteins from *B*. *subtilis*. The previous structural studies of *E*. *coil* YhdE (Fig 1) explained a possible molecular mechanism and specificity of PPase activity [5]. The overall structure of YhdE is composed of two lobes with a cleft formed in the center. Interestingly, the two structures of YhdE from the different crystal forms displayed substantially different conformations at the cleft consisting of R13, K82, K146, E32, E81 and other related residues where the active site is located. The structure of the apo form (P4 space group) showed an ‘open’ conformation, while the structure from the second crystal form (P2_1_2_1_2_1_ space group) displayed a ‘closed’ conformation [8]. The major difference between the two conformers is reflected in the shape and volume at the pocket of the active site cleft and its surface charge distribution. For example, residue E32 in loop-4 (Table 1) can adopt two alternative conformations with its carboxyl group pointing in and out of the substrate binding pocket with a 5.72 Å-shift between the open and closed conformations. The underlying implication for the existence of two states is unclear. Also, in our previous enzyme activity study [8], the PPase activity of YhdE was cooperative, suggesting that there are possible intermolecular interactions between two YhdE molecules as YhdE are dimers in both crystal forms.

**Table 1:** Secondary structure statistics of YhdE in both open and closed states.

Secondary Structure Identifier	Residue Range	Secondary Structure Identifier	Residue Range
Loop-1	1	Strand-5(β5)	112~124
Strand-1(β1)	2~4	Loop-9	125~128
Loop-2	5~8	Helix-4(α4)	129~138
Helix-1(α1)	9~18	Loop-10	139
Loop-3	19~21	Helix-5(α5)	140~143
Strand-2(β2)	22~24	Loop-11	144~152
Loop-4	25~38	Helix-6(α6)	153~157
Helix-2(α2)	39~57	Loop-12	158
Loop-5	58~63	Strand-6(β6)	159~162
Strand-3(β3)	64~72	Loop-13	163~164
Loop-6	73~83	Helix-7(α7)	165~169
Helix-3(α3)	84~94	Loop-14	170~172
Loop-7	95~97	Helix-8(α8)	173~188
Strand-4(β4)	98~109	Loop-15	189~190
Loop-8	110~111		

Prefix “β” indicates β-strand and “α” indicates α-helix.

Thus far, the molecular mechanisms for nucleotide binding, catalysis and cooperative effects of YhdE have been not identified except for the recent paper describing the PPase activity of YhdE toward canonical and modified nucleotides [5]. In order to further investigate the stability for both open and closed conformations of YhdE as well as the cooperative effects for YhdE PPase activity, we have performed crystallization experiments and molecular dynamics studies based on the open/closed conformations. The intermolecular interactions between two YhdE have also been examined.

## Methods and Approaches

### Crystallizations

In our previous structural studies, the inactive YhdE-E33A mutant designed to promote substrate bound complex formation was used for crystallization in the presence of dTTP substrate [8]. In this work we carried out crystallization in the absence of dTTP. In general, the preliminary crystallization conditions for the YhdE sample were screened by the sparse matrix method using standard screening kits. A wide range of protein concentrations was tested, ranging from to 0.5 to ~10 mg/mL in 20 mM Tris (pH 8.0) and 30 mM NaCl. The hanging drop vapor diffusion method was used; hanging drops were set up with 2 μL of protein solution mixed with 2 μL of well solution. The optimal crystallization condition in the reservoir was 0.1 M MgSO_4_, 0.1 M MES buffer (pH 6.5), and with 20–30% PEG4000 as the precipitating agent at room temperature.

### Molecular docking and model building

All molecular dockings were accomplished using Autodock [9]. Amino acids involved in the active site of YhdE were set as flexible residues. Genetic Algorithm was used to minimize system energy and prepare docking conformations. For each docking, 100 possible substrate conformations were exported for clustering analysis. Conformations with minimum energy were used for the subsequent molecular dynamics (MD) simulations. Model buildings for the next simulation step are shown in Table 2.

**Table 2:** Models used in molecular dynamic simulations.

Model Name	Protein	Substrate (dTTP)
Model 1	open state dimer	(no substrate)
Model 2	closed state dimer	(no substrate)
Model 3	closed state monomer	docking result
Model 4	open state monomer	same as in Model 3
Model 5	open state dimer	same as in Model 3
Model 6	open state dimer	same as in Model 3
Model 7	closed state dimer	same as in Model 3
Model 8	closed state dimer	same as in Model 3

The “docking result” in Model 3 indicates that dTTP conformation with the lowest docking energy and largest clustering number are extracted for further calculation. dTTP conformation in other models are set by superimposition.

### Molecular dynamics simulation

All MD simulations were carried out using the AMBER 12 package [10] and amber2010 all atom force field parameters [11] together with General Amber Force Field (gaff) parameters [12,13]. 4~7 Na^+^ ions were introduced to neutralize charge. Each system was explicitly solvated by using the TIP3P water potential inside a box of water molecules with a minimum solute-wall distance of 10 Å. The protocol for all MD simulations was as follows: (1) the whole system was energy minimized to remove unfavorable contacts. Four rounds of 2500-steps minimization were performed. In the first two rounds the whole system was restrained except water and Na^+^ ions; minimization methods used in the first two rounds were Steepest Descent and Conjugate Gradient, respectively; in the last two rounds the whole system was unrestrained and minimization methods used in the first two rounds were Steepest Descent and Conjugate Gradient, respectively. The cut-off distance used for the non-bonded interactions was 10 Å. SHAKE algorithm was used to restrain the bonds containing hydrogen atoms; (2) the energy-minimized structure was heated over 200 ps from 0 to 300 K (with a temperature coupling of 0.2 ps), while the atom positions of protein were restrained with a small value of 10 kcal/(mol Å^2^); (3) the unrestrained equilibration of 200 ps was carried out for each system in NPT ensemble with the temperature and pressure of 300 K and 1 bar, respectively, with the corresponding coupling of 0.2 ps. An integration step of 2 fs was used; and (4) finally, 50 ns unrestrained molecular dynamics was carried out for the protein model. Other simulations followed the same protocol; (5) for the conformation output step, the average frame of last 10 ns trajectory was exported for overall structure analysis.

### Fluctuation and correlation analyses

The root-mean-square fluctuation (RMSF) values of residues are a measurement of fluctuations and flexibilities of backbone Cα atoms of protein over the trajectory which were broken down by residues in comparison with the average structures. RMSF_i_ of the Cα atom of each residue was calculated as follows:

$$RMSF_{i}=\sqrt{\frac{\sum_{t=1}^{T}{\left(r_{i}\left(t\right)-\langle r_{i}\rangle \right)}^{2}}{T}}$$

Where T is the number of snapshots considered in the time trajectory, *r*_*i*_(*t*) the position of the Cα atom of residue i at time t, and $\langle r_{i}\rangle$, the time-averaged position of the Cα atom of residue i.

The dynamic feature of a protein and extent of correlation of motions in different regions of a protein were assessed via the calculation of cross-correlation coefficients, or C(i,j), as follows:

$$\text{C}\left(\text{i},\text{j}\right)=\frac{\left(\Delta r_{i}\times \Delta r_{j}\right)}{\sqrt{\left(\Delta r_{i}{}^{2}\Delta r_{j}{}^{2}\right)}}$$

Where Δr_*i*_ and Δr_*j*_ are the displacement vectors for Cα atoms of residues i and j, respectively; and the angle brackets denote the ensemble averages. In the present study, the correlation coefficients were averaged over the regions of protein, and the resulting cross-correlation coefficients are presented in the form of a two-dimensional graph [14]. These structure analyses in the present work were performed using the PTRAJ module of the AMBER 12 program [10].

### Binding pocket and free energy analyses

The Computed Atlas of Surface Topography of proteins (CASTp) program was used to determine the substrate binding pocket [15]. The CASTp program uses weighted Delaunay triangulation and the alpha complex for shape measurements. Protocols used in this study were consistent with our previous work [16].

For the free-energy analysis, the molecular mechanics Possion-Blotzmann surface area (MM-PBSA) method in AMBER 12 package was employed [17–19]. The binding free energy was obtained through calculating the free energy differences of ligand, receptor, and their complex as follows:

$$\Delta {\text{G}}_{binding}=G_{complex}-G_{ligand}-G_{receptor}$$

In MM-PBSA, the free energy (G) of each state is estimated from molecular mechanical energy *E*_*MM*_, solvation free energy *G*_*solv*_ and vibrational, rotational, and translational entropies *S*, respectively. Computational details were similar to our previous studies [20,21]. It is well known that MM-PBSA free energies do not usually replicate the experimental free energy in absolute value. This approach calculates binding affinity ranking and exhibits good correlation with experiments. Therefore, MM-PBSA only provides modest accuracy for relative binding affinities in systems dominated by electrostatics.

## Results

### Crystallization experiments show that the closed state is a stable conformation in the absence of dTTP

A wide range of protein concentrations and crystallization conditions were tested. We obtained two crystal forms, similar to the previous reported crystal forms [8] of two space groups, namely P4 and P2_1_2_1_2_1_. Our results show that 0.5–1 mg/mL YhdE sample forms crystals of P4 space group which represents the open state [8]. At >5 mg/ml, YhdE forms crystals with P2_1_2_1_2_1_ space group corresponding to the closed conformation. This observation suggests that closed state could exist in the absence of substrate dTTP, indicating that YhdE closed state itself is a stable conformation.

### Molecular docking and dynamics simulations

**Fig 2:**
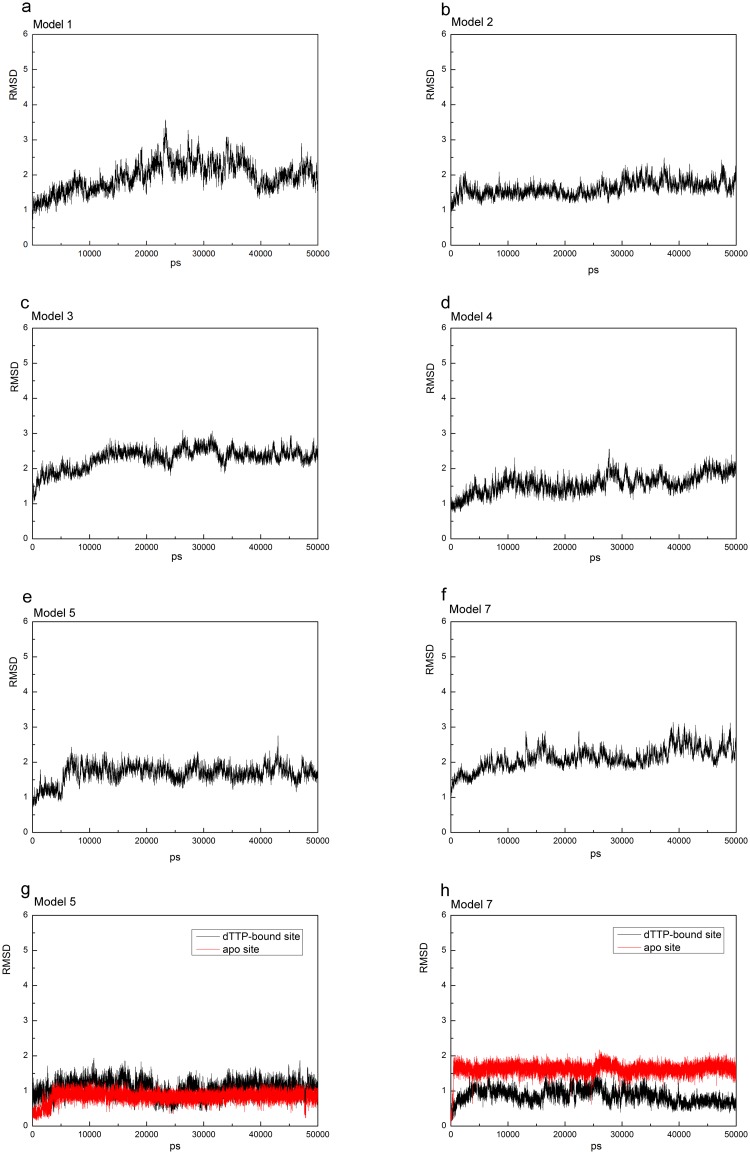
RMSD of YhdE-dTTP complex in each models and RMSD of loop-6 in model 5(g) and model 7(h). Model 5 and Model 6 are the same simulating model with different receptor/ligand combination (see Table 2), so as to Model 7 and Model 8.

To further investigate stability and conformational differences between YhdE open and closed states, we performed molecular dynamics simulation of the two YhdE structures (PDB codes: 4P0U and 4P0E) in both monomer and dimer forms. All models reached equilibrium after 30 ns, and their energies were stable during the remaining simulations (data not shown). Thereafter, for each system, the trajectory analysis was performed to extract the equilibrated conformation between 40 and 50 ns of the simulation time, recording 5000 snapshots (one for each 2 fs). The RMSD (root mean square derivations) variation analyses for each model are shown (Fig 2). The RMSDs of all backbone atoms between each frame and original crystal structure are all lower than 3 Å, indicating that both states are sufficiently stable for analyzing the residue interactions.

**Fig 3:**
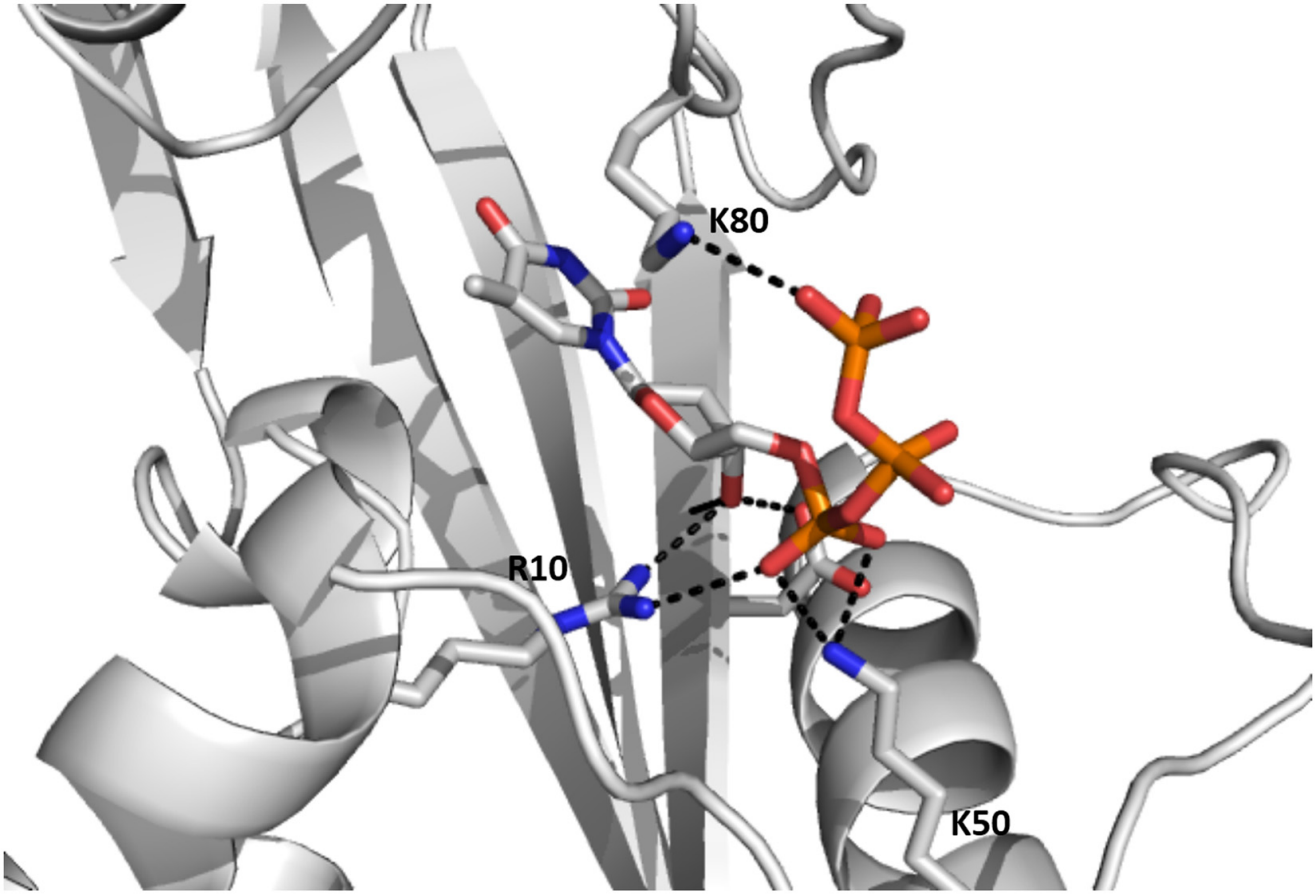
Molecular dynamics simulation result of YhdE open state with dTTP.

**Fig 4:**
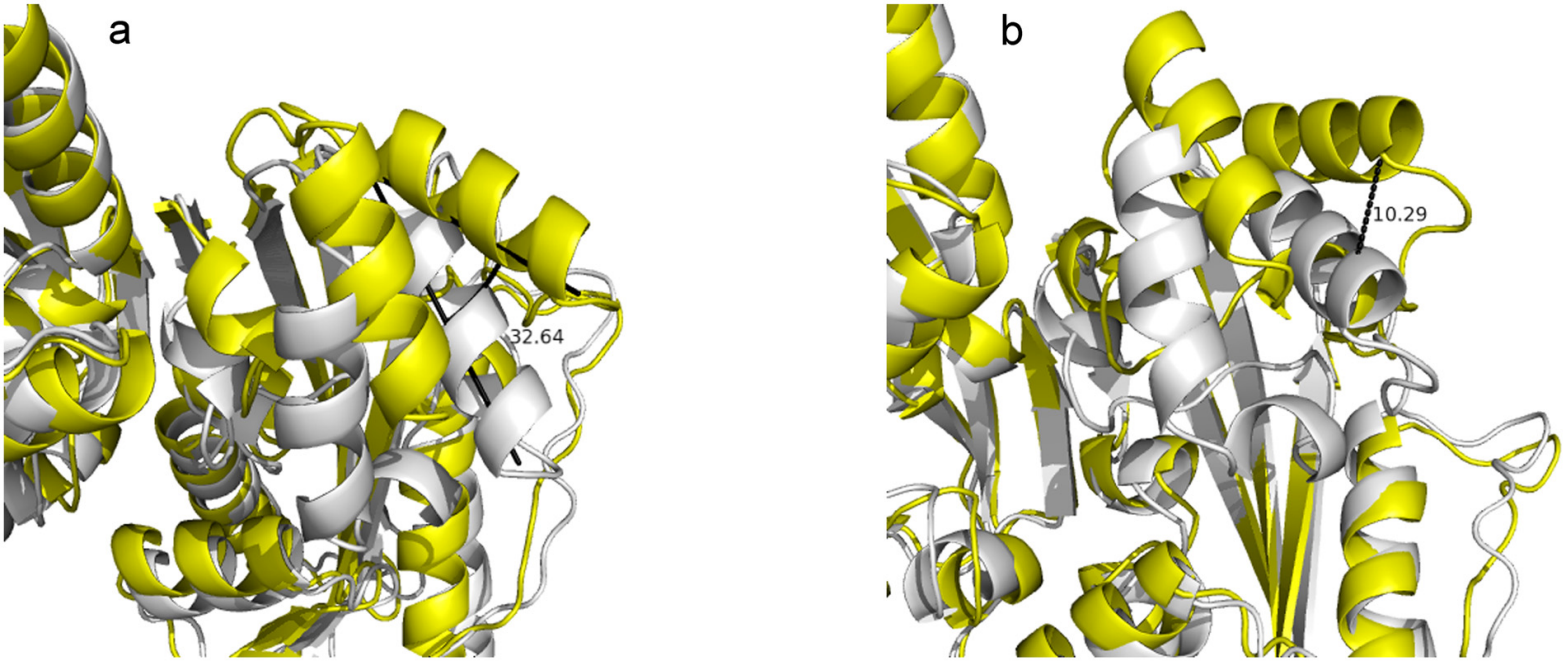
The alignment of the apo-open state dimer (average structure, yellow) and the closed state dimer (average structure, white). Distance between the two catalytic loops in each conformation is shown in Fig 4a in Å and twisting angle is shown in Fig 4b in degree.

Since there was no dTTP molecule in the structure of YhdE co-crystallized with dTTP [8], we performed molecular dockings of dTTP into both open and closed states of YhdE and carried out molecular dynamics simulation to examine their stability. In dTTP-bound forms of YhdE we modelled, dTTP behaved quite differently in YhdE monomer compared to YhdE dimer. Average structure analysis shows that dTTP base group was less stable in YhdE monomer in both open state and closed state, which suggests that the YhdE monomer is less suitable for substrate stabilization. However the phosphate groups of dTTP in all dTTP-bound state is quite stable in average structure (Fig 3). To ensure that the start conformation of dTTP did not affect final results, dTTP conformation in closed state was extracted and superimposed to the open state to perform MD simulation again with the same parameters (Model 4). RMSD result shows that the overall structure of the substrate bound form in the closed state was generally stable and only a slight increase of RMSD was observed. Here all phosphate groups remain in the same conformation as in the dTTP-docked YhdE of the closed state (Model 3). These results indicate that residues I151, R10, R11 and K50 could stabilize dTTP without the help of catalytic loop (loop-4 in Table 2). dTTP in the closed state is quite stable during the entire molecular dynamics simulation. In the average dimer structure of the apo-closed state, helix-3, helix-4 and loop-6 have a 10.36 Å movement and a 32.6° turning in comparison with the apo-open state (Fig 4). While in the dTTP-bound form, this structural variation could not be detected when the open state was aligned to the closed state, and vice versa. It indicates that the closed state is a high-tension structure at room temperature and may squeeze dTTP or other substrates with residues in helix-3, helix-4 and loop-6. This squeezing affect could reflect that closed state help YhdE reaction from structural aspect.

**Fig 5:**
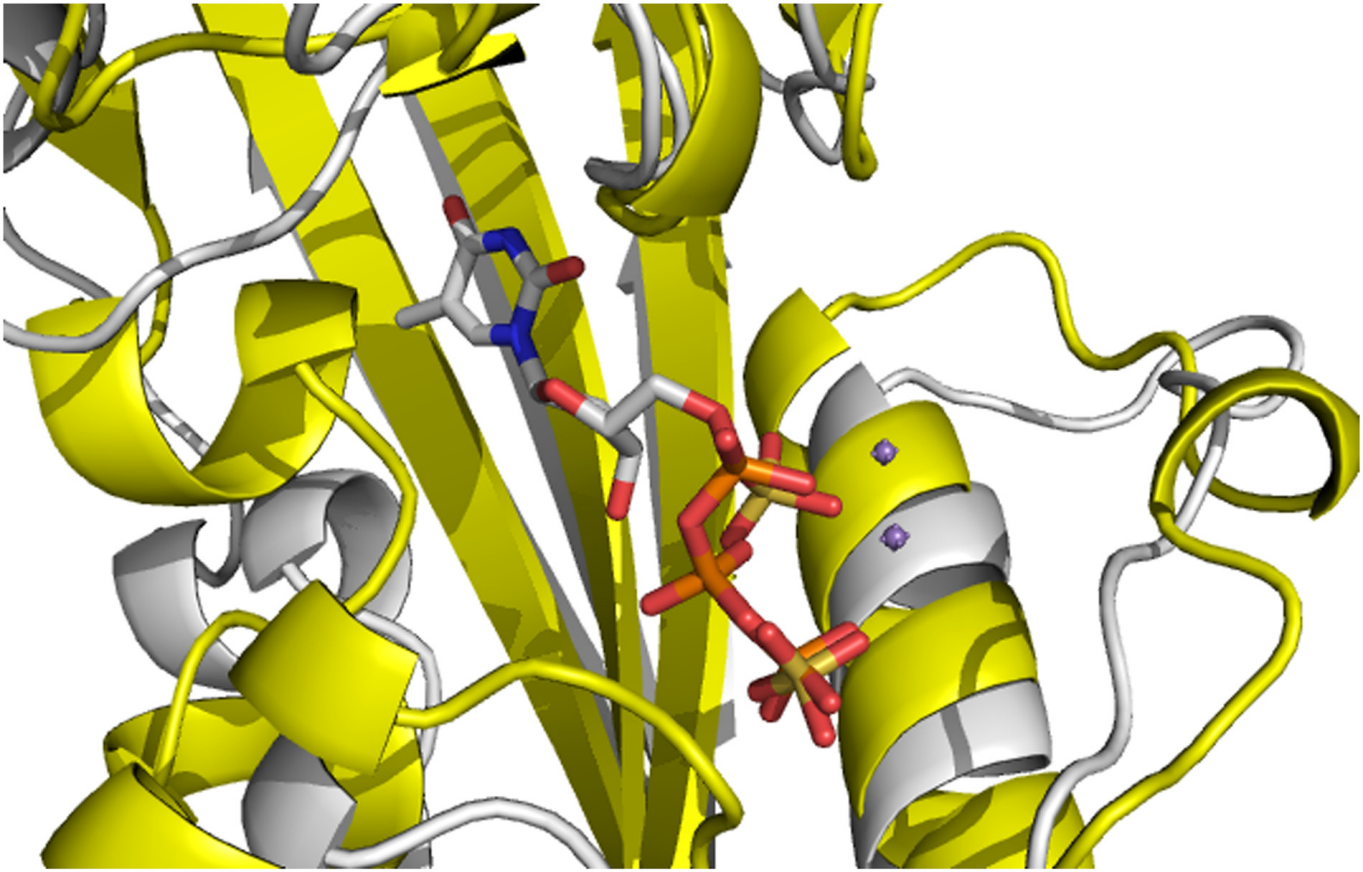
Alignment of the average dTTP-bound closed state structure (white) with the Tbru21784AAA structure (yellow). Sulfate groups and Mn^2+^ ions are shown in sticks and brown spheres, respectively. dTTP is shown in sticks using the same color.

This substrate binding model is in good agreement with the crystal structures of YhdE homologs. In the crystal structure of *T*. *brucei* Tbru21784AAA (also a Maf-like protein, PDB code: 2AMH), two sulfate ions are observed in the same positions where α-phosphate and γ-phosphate of our dTTP docking are located (Fig 5). Moreover, in the MD simulation two sodium ions are found in the same place where two manganese ions are located in Tbru21784AAA structure. The E45 residue in Tbru21784AAA takes almost the same conformation as the equivalent residue E32 in YhdE. This agreement confirms that our dTTP-bound YhdE model is reasonable for further structural and molecular dynamic characterization. Under this consideration, it was predicted that R13, K82, K146, E32, E81 would be critical for dTTP binding.

**Fig 6:**
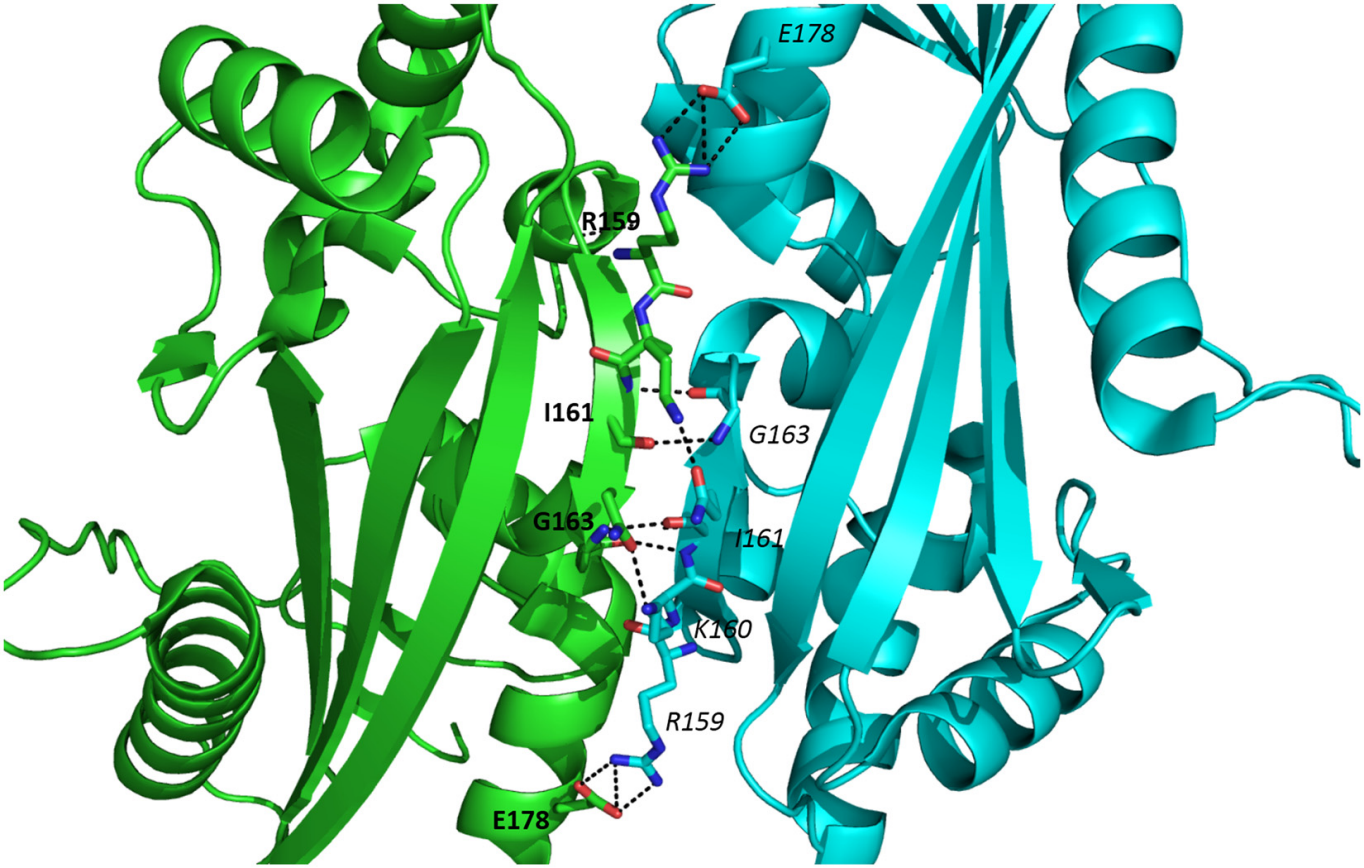
Intermolecular interactions between two β6 strands in YhdE dimer. Residues in two YhdE molecules which are involved in intermolecular β-strand are presented with bold and italic, respectively.

Further, it is known that YhdE is able to form an intermolecular β-strand between two β6 strands of YhdE dimer (Fig 6). Our pervious size-exclusion chromatographic result showed that YhdE forms dimer in solution and enzyme activity experiment indicated that dTTP hydrolysis reaction catalyzed by YhdE displayed significant cooperative effect [8], raising a question whether dimerization would affect YhdE catalytic reaction.

### Free energy analyses reveal that intermolecular β-strand interaction forms first

To address whether the positive cooperative effect of the dTTP binding to YhdE is induced by YhdE dimerization, or *vice versa*, the binding free energy calculations for one YhdE molecule binding to apo-form YhdE molecule or binding to dTTP-bound YhdE molecule were carried out using MM-PBSA method based on MD simulations. The free energy calculations for dTTP bound YhdE monomer and dimer were also carried out. All energy terms and the total binding free energies for these systems are shown in Table 3. It shows that YhdE monomer binding to the apo-form YhdE has the binding energy of -152.27 kcal/mol, compared to its binding to the dTTP-form which is -132.11 kcal/mol, it also suggests that dTTP binding to YhdE monomer is a little less favorable than binding to YhdE dimer, with binding energies of -71.36 kcal/mol and -82.06 kcal/mol, respectively. The free-energy reduction of YhdE dimerization is much more (>120kcal/mol) than that dTTP binding with YhdE (70kcal/mol~80kcal/mol), indicating that YhdE binding to another YhdE is energetically more favorable than YhdE binding to dTTP. Despite of the expected error in the free energy calculation, this energy ranking may indicate that YhdE dimerization benefits dTTP binding. The calculated binding free energies of these systems support the corresponding experimental results in the previous studies [5,8].

**Table 3:** MM-PBSA Free Energy Components for each models.

	Model 1	Model 2	Model 3	Model 4	Model 5	Model 6	Model 7	Model 8
**receptor**	apo-YhdE	apo-YhdE	YhdE	YhdE	YhdE-dTTP	YhdE-dimer	YhdE-dTTP	YhdE-dimer
**ligand**	apo-YhdE	apo-YhdE	dTTP	dTTP	apo-YhdE	dTTP	apo-YhdE	dTTP
***ΔE**_**ele**_*	-255.58	-226.38	-208.65	-280.69	-206.98	-177.11	-57.64	-99.92
***ΔE**_**vdw**_*	-108.11	-95.37	-36.20	-30.60	-97.05	-34.64	-98.85	-36.52
***ΔE**_**pb**_*	286.10	250.26	235.72	264.86	242.58	152.70	91.17	84.51
***ΔE**_**gb**_*	325.17	293.64	226.12	283.27	271.59	175.25	121.39	97.54
***ΔE**_**np**_*	-74.68	-69.72	-28.02	-24.94	-70.66	-28.93	-72.50	-29.61
***ΔG**_**gas**_*	-363.69	-321.76	-244.26	-311.29	-304.03	-211.76	-157.49	-136.44
***ΔG**_**solv**_*	211.47	180.54	207.69	239.92	171.92	123.77	18.66	54.90
***ΔTS***	-60.30	-60.14	-44.21	-37.10	-60.09	-37.68	-60.54	-38.07
***ΔG**_**binding**_*	-152.27	-141.21	-36.57	-71.36	-132.11	-87.99	-138.83	-81.54

All values are in kcal/mol.

**Fig 7:**
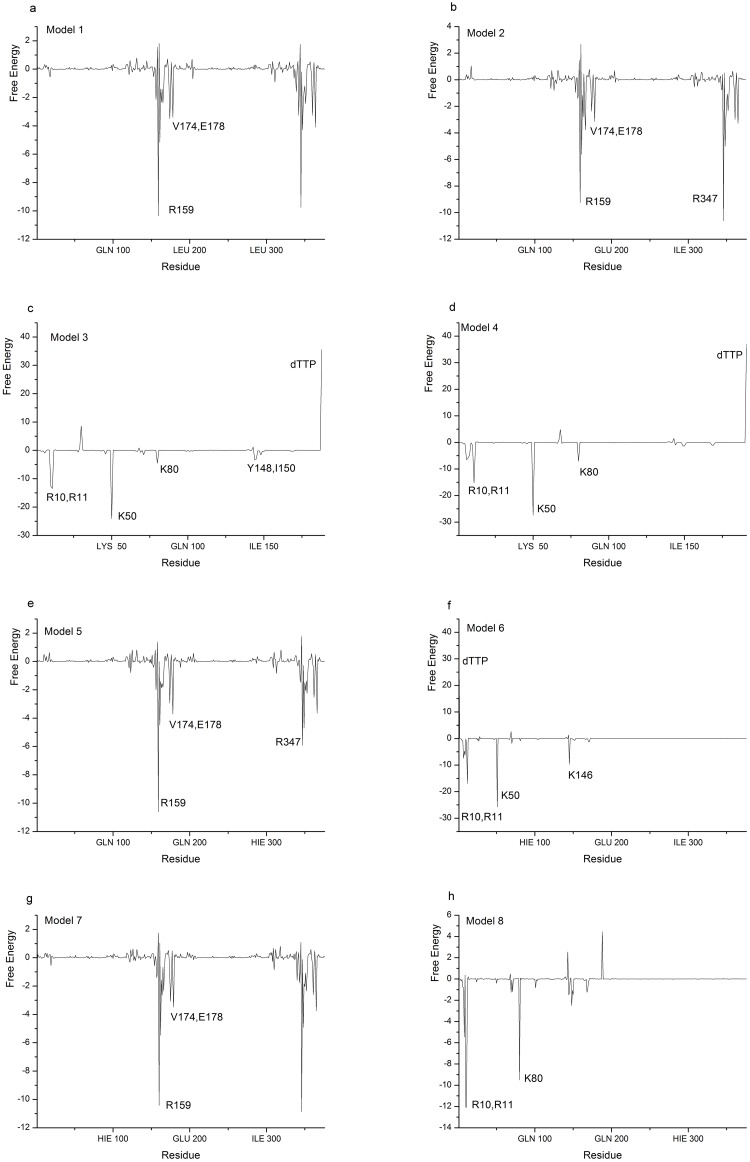
MM-PBSA energy decompositions (kcal/mol) into the residues of each models. The key residues in each model are labeled.

To further understand the role of each residue in dTTP binding in YhdE dimer, decompositions of the corresponding binding free energy into the residues of YhdE in the dTTP-bound YhdE dimers were performed. Results are shown in Fig 7. The decompositions of binding energy provided more quantitative information of energy contribution of each residue. It can be seen that the distinct differences of binding free energy decompositions between one YhdE molecule binding to apo-YhdE monomer and to dTTP-bound YhdE monomer models mainly occur at the binding sites of dTTP binding site, namely, at the residues R10, R11, K50, K80, V174, V178, R159. The binding free energy are largely contributed by these residues, indicating these residues are of great importance. These analyses also suggest that in dimerization processes for both open and closed state in their apo-form, free energy distributions are almost the same in each residues. Analyses for Model 3 and Model 4 indicate that both YhdE open and closed states mainly interact with dTTP (conformation in closed state) through R10, R11, K50 and K80 from energy’s point of view. However analysis of Model 6 shows that K80 became less important in YhdE open state dimer that interacts with dTTP in comparison with Model 4. Analysis of Mode 8 shows that R10, R11 and K80 became more important but K50 lost its importance.

### Polar contacts analysis of dTTP binding to YhdE and intermolecular β-strand in YhdE dimer

**Fig 8:**
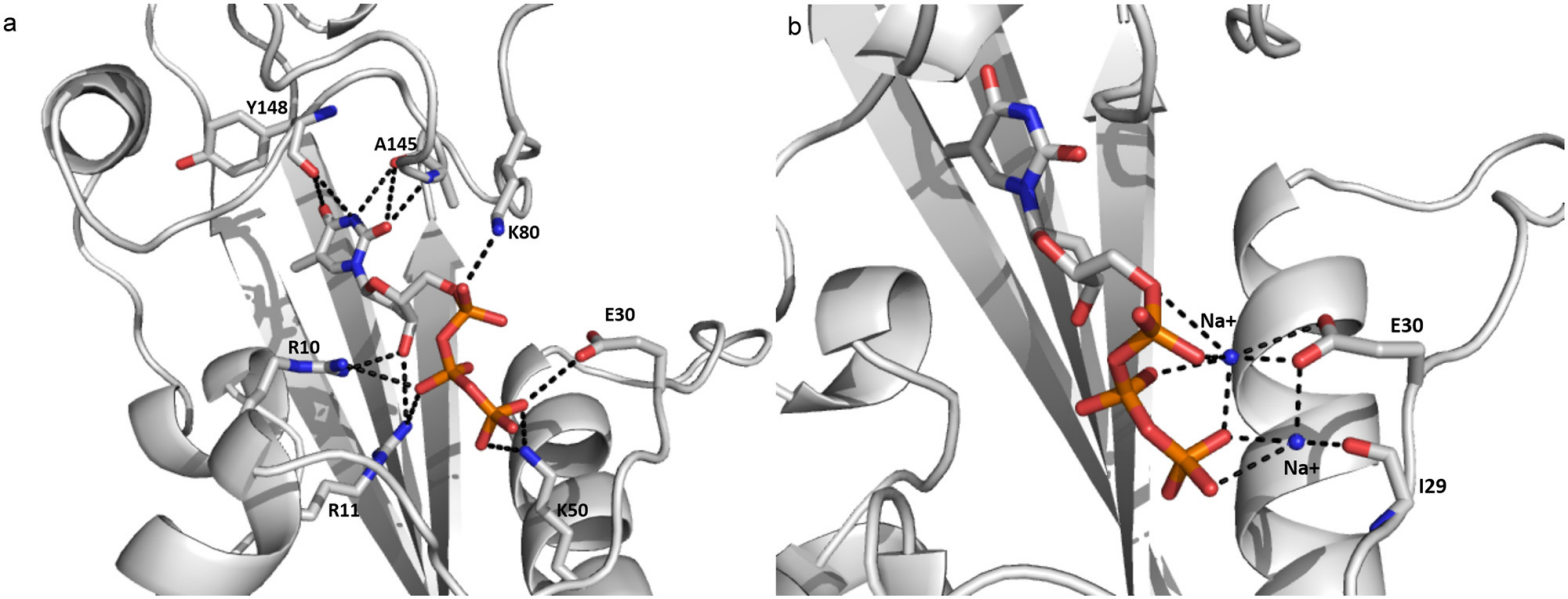
Average conformation of the YhdE closed state with dTTP bound in MD simulation (a) and the YhdE open state with dTTP bound in MD simulation (b). The diagram on the left shows the interactions between dTTP and YhdE residues in the binding pocket. The diagram on the right shows two sodium ions that interact with dTTP and I29, E30 at the same time in the MD simulation.

Free energy studies on YhdE open/closed states with dTTP provided information about previously interactions between substrate dTTP and residues of YhdE. However such calculations could not distinguish the contributions of polar contacts from non-polar ones. To gain information of the polar contacts, including hydrogen bonds and electrostatic interactions of dTTP-bound YhdE in the closed state, trajectory analysis of hydrogen bonds and electrostatic contacts were performed [22,23]. The occurrences of all possible hydrogen bonds between dTTP and side chains of residues the binding pocket are shown by calculating the percentages of times in the MD simulations. The criteria for a hydrogen bond was a donor-acceptor distance of <3.5Å and a donor-proton-acceptor angle of >120°. Table 4 shows the polar contacts around dTTP. Contacts in the dTTP-bound YhdE open state are also shown as a comparison. There are some varied hydrogen bonds between dTTP and open/closed state of YhdE, among which hydrogen bonds between K50 and dTTP changes significantly from Model 3 to Model 4 as well as from Model 6 to Model 8. The hydrogen bonds are weak in Model 3 and Model 8 than those in Model 4 and Model 6. It suggests that K50 is less important in dTTP binding to YhdE-dimer in both open/closed states, which is consistent with the free energy analysis result. Similarly, it shows K80 is more important in dTTP binding to YhdE closed state than in dTTP binding to YhdE open state. These hydrogen bond statistics have provided detailed information about interaction variations in dTTP-YhdE interaction. Average structure of MD simulations in both dTTP-bound states shows that in the open state, dTTP binding is highly unstable in comparison with that in closed state, which is reflected by a mass average conformation of dTTP. The average conformation of dTTP binding in the closed state of YhdE is shown in Fig 8. This conformation indicates that K80 in loop-6; E30 in loop-4 (catalytic loop), R10, R11 in loop-2; and K50 in helix-2 play important roles in substrate stabilization. Interactions between dTTP and K50/A145 are considerably increased by >90%, which contributes most to the stabilization of dTTP phosphate groups. Additionally, the hydrophobic interaction between the phenyl group of dTTP and surface formed by β3~β6 strands was detected with >90% occupancy of the simulation time for the dTTP-bound closed state of YhdE. These interactions predict that the cooperative effect of YhdE dimerization enhances dTTP binding affinity, which is consistent with the energy decomposition results discussed above. It is worth noting that negative charged side chains repulse phosphate groups of dTTP as they carry negative charge. Distance between the side chains and dTTP is less than 4 Å, which indicates that hydrogen bond between dTTP and YhdE side chain residues is much stronger than electrostatic repulsion.

**Table 4:** Occupancies (%) of Hydrogen bonds between dTTP and YhdE in model 3, 4, 6 and 8.

Hydrogen bond	Model3	Model4	Model6	Model8
(dTTP)O9-H-NH2(11)	5.41	73.48	3.62	0.21
(dTTP)O11-H-N(150)	90.83	28.52	97.94	88.92
(143)O-H-NZ(80)	13.4	38.74	20.34	21.42
(dTTP)-O12-H-NZ(80)	52.06	0	0	6.86
(dTTP)O14-H-NZ(50)	48.95	97.3	99.88	24.37
(dTTP)O12-H-NZ(50)	39.4	94.88	49.56	0.14
(dTTP)O10-H-N(145)	64.04	91.76	8.66	93.21
(dTTP)O5-H-NH2(11)	0	43.62	0	88.66
(145)O-H-N2(dTTP)	0	39.64	0	0
(143)O-H-NZ(80)	0	38.74	20.63	24.73
(dTTP)O2-H-NZ(80)	51.73	31.23	14.65	36.89

A hydrogen bond is listed in this table only if its occupancy is >30% in at least one model.

### Accessible areas (pockets) in YhdE crystal structures and MD average structures

**Fig 9:**
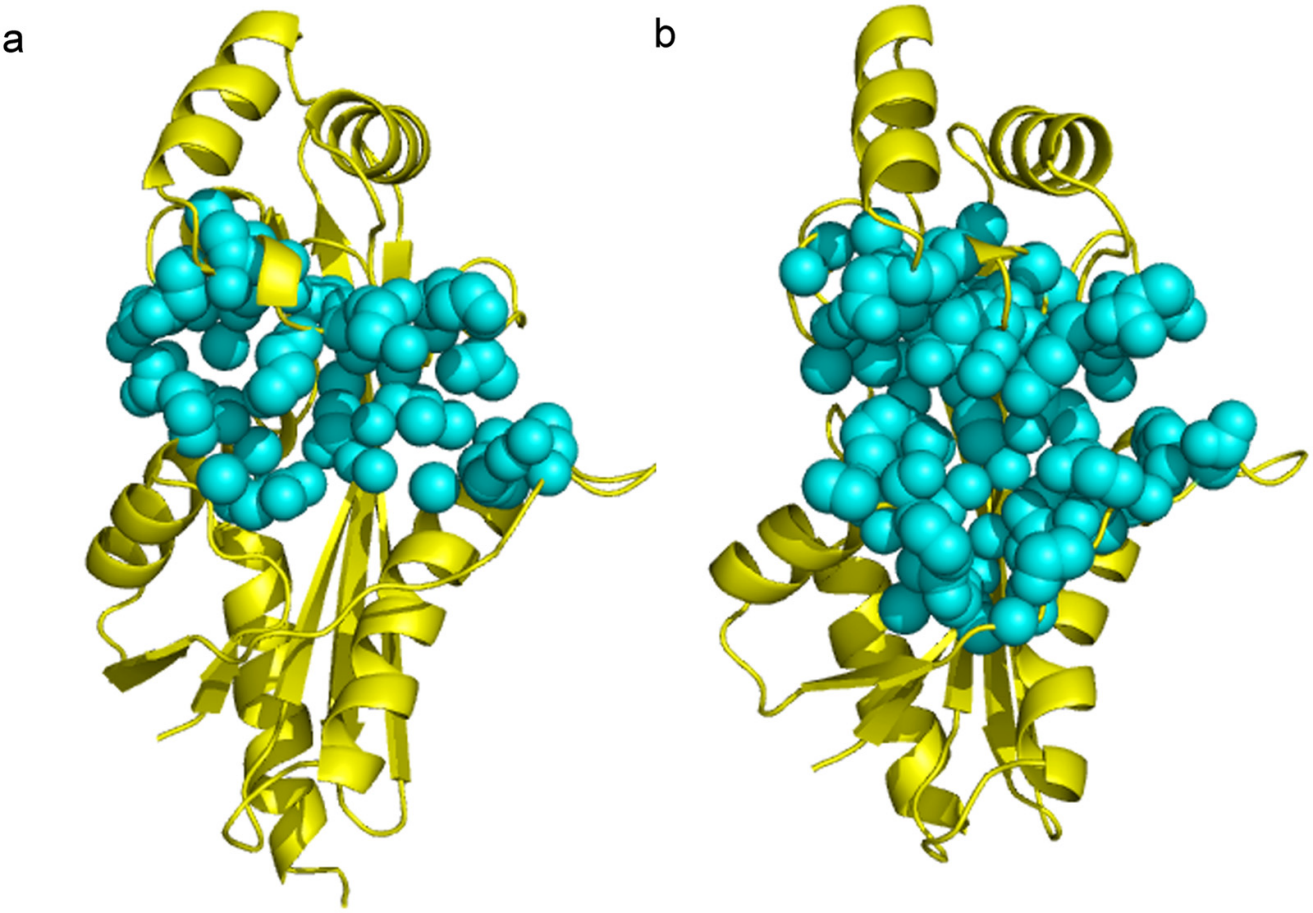
Binding pocket analysis of YhdE in the open state (a) and in the closed state (b). The binding pocket in each structure is shown as cyan sphere.

Conformational variations in MD simulation lead the allosteric process of the regulatory loop, the β6 strand, and further affect the accessible area of the active site. To address the exposed space of the active site, accessibility of the binding pockets in the crystal structure [8] and in MD simulation was calculated. Fig 9 shows the pockets in the open and closed states, which are automatically identified by the CASTp analysis (see Methods and Approaches). The topological cavities in the open state and close state reveal different “mouth” area (defined as in CASTp program) for the active site pockets, to be 342.15 Å^2^ and 308.71 Å^2^, respectively. The lengths of the “mouth” areas for the active pockets in the open and close states are 155.69 Å and 145.68 Å, respectively. The measurements of “mouth” area reflect the variation of open and closed state of YhdE. Accessible areas of the active site are 728.71 Å^2^ and 1268.18 Å^2^ in the open state and closed state, respectively; and volumes of the active site are 1358.38 Å^3^ and 1974.19 Å^3^ in open state and closed state, respectively. Measurements for the MD average structure of the open/closed states are consistent with the crystal structure.

The differences of the area and volume at the active site between the open state to closed state show that the size of active site in the closed state is larger than the open state while only small variation in “mouth” area could be detected, suggesting that the closed state tightens the entrance for substrate dTTP and gives more space for dTTP binding within the active site area. It would facilitate catalytic reactions since the closed state is a more favorable conformation for substrate binding and is a slightly less favorable conformation for substrate releasing. It could explain the stability variation between the dTTP-bound open state and dTTP-bound closed state of YhdE. Moreover, the calculations of the MD simulation average structure (Table 5) suggest that the closed state dimer possesses an asymmetry conformation in which dTTP could only bind to active site one by one. This asymmetry reflects the tension lies in the YhdE closed state and indicates that YhdE catalyzed dTTP hydrolysis through a mechanism in which one dTTP binds to one active pocket in YhdE dimer at the same time.

**Table 5:** Accessible area calculation of each average structure in MD the simulation.

	Accessible Pocket Area (Å^2^)	Accessible Pocket Volume (Å^3^)	Accessible Mouth Area (Å^2^)	Accessible Mouth Length (Å)
open-apo-dimer	775.88/778.05	1375.01/1455.60	288.71/313.88	127.92/117.12
closed-apo-dimer	429.35/624.74	804.41/908.57	201.77/180.96	84.40/107.98
open-dTTP-dimer	522.66	1127.68	341.90	123.71
closed-dTTP-dimer	896.53	1174.87	437.05	227.74
open-apo-monomer	728.71	1358.38	342.15	155.69
closed-apo-monomer	1268.18	1974.19	308.71	145.68

Open/closed apo dimer contains two active pockets and are listed as “first pocket/second pocket” in the table.

### Structural characteristics of YhdE conformational change induced by dTTP bindings

**Fig 10:**
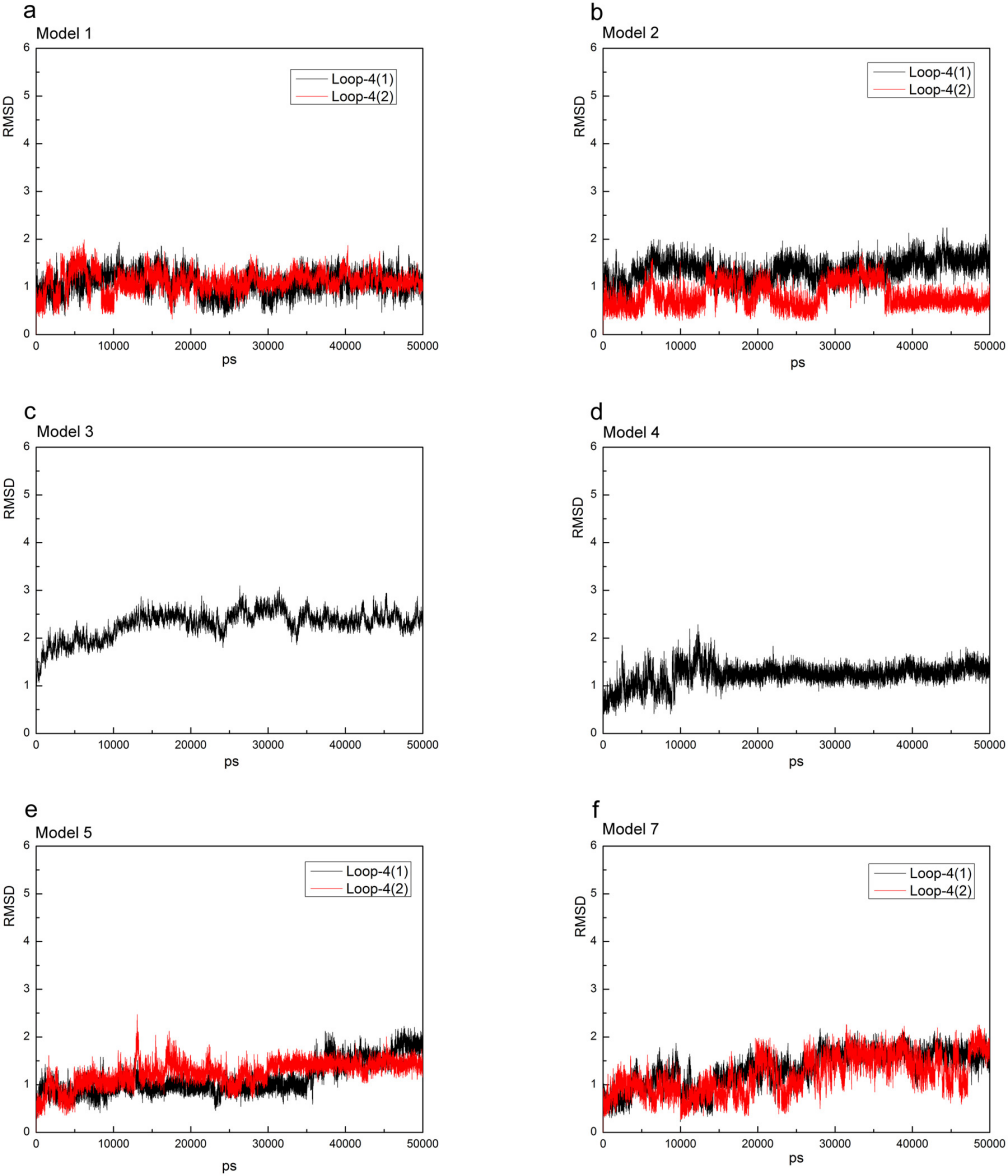
RMSD vs. simulation time of the catalytic loop (loop-4) in each model. Loop-4-1 and loop-4-2 represent two loop-4s in the model because of two YhdE molecules. RMSD vs. time of loop-4 in the apo-YhdE monomer of open/closed state is shown for comparison.

To address the structural variations of catalytic loop (loop-4) in YhdE with open/closed conformations, the average structures from the equilibrated simulations of the apo open state and closed state of YhdE are shown in Fig 10. All these RMSD values are lower than 3 Å, suggesting that loop-4 in both states and in both monomer/dimer forms is stable. Overall structure rotations are tested and measured by using the DynDom program (http://www.cmp.uea.ac.uk/dyndom/). The relative rotation angle variation between the two states is very small. The largest is only 1.42° rotation which is close to the difference between the two crystal structures (1.63°), suggesting that there is no significant structure rotation between the open and closed state in every models. However, helix-3, helix-4 and loop-6 in YhdE show large conformational variations between the open state and closed state (Fig 4). Such variation suggests that the closed state is theoretically a more tight conformation than the open state. It is consistent with our previous suggestions that the catalytic loop is pushed closer to the active site, facilitating the reactions. Loop-2 is actually more important than loop-4 from the average structure analyses of each models, as there are more critical residues interacting with dTTP. In the dTTP-bound form of YhdE closed states dimer, loop-2 shows significantly different RMSD values. These RMSDs show that loop-2 in two YhdE molecules behave almost the same in the dTTP-bound open state dimer, indicating that loop-2 does not closely interact with dTTP in the dTTP-bound site. However in the dTTP-bound closed state, RMSD of loop-2 in the dTTP-bound site is significantly lower than loop-2 in the apo site. This variation suggests that in the dTTP-bound closed state, dTTP is squeezed by helix-3, helix-4 and loop-6 so that dTTP closely interacts with residues in loop-2 and nearby residues, which may promote the catalytic reaction.

The average structure of the closed state with dTTP bound from our MD simulation also suggests that the phosphate groups interact with YhdE. The β-phosphate and γ-phosphate groups are found closely interacting with positive charge residues (R10, R11) in the average structure of the dTTP-bound closed state (Fig 5). This model with positive charge residues interacting with the central phosphate is commonly seen in protein kinases, in which arginine or lysine often acts as an electron dragger and interacts with γ-phosphate only. This model provides a possible suggestion for the pyro-phosphatase activity of YhdE. Under the squeezing by helix-3, helix-4 and loop-6, bond between α-phosphate and β-phosphate is broken with the help of R10, R11 and other positive charged residues, causing dTTP hydrolysis.

**Fig 11:**
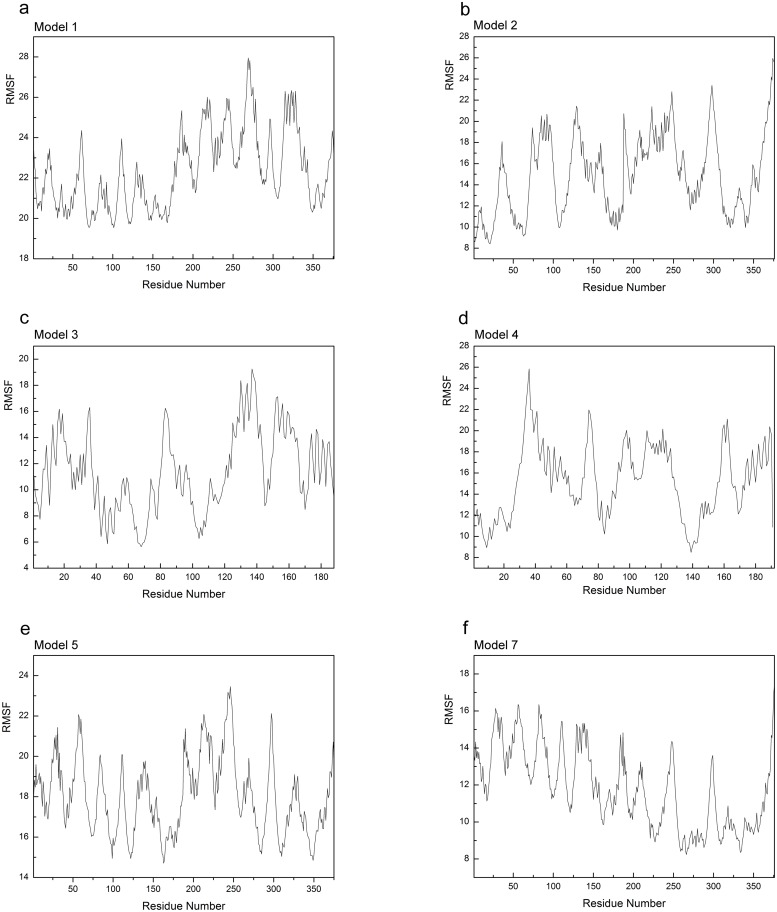
RMSF values (Å) of each models. Apo-YhdE monomers in both open and closed state are shown for comparison.

RMSF variation between the apo-open state and the closed state of YhdE (Fig 11) or between the dTTP-bound open state and the dTTP-bound closed state reflects the flexibility of residues in each state. It shows that in the closed state, the catalytic loop becomes more closely packed than in the open state, no matter in apo form or in dTTP-bound form, which is consistent with the observations above and suggests that YhdE closed state represents the reaction state in which the substrate is stabilized by the catalytic loop with the allosteric commutations from nearby helixes or strands. However in Model 1 and Model 2 only one YhdE molecule shows large RMSF value, suggesting that YhdE dimerization restraints one of its component. In Models 3, 4, 5 and 7, RMSF significantly decreases, indicating that dTTP binding reduced flexibly of YhdE in both YhdE monomer and dimer.

### Allosteric communication study by correlation factor analysis in the open/closed state of YhdE

**Fig 12:**
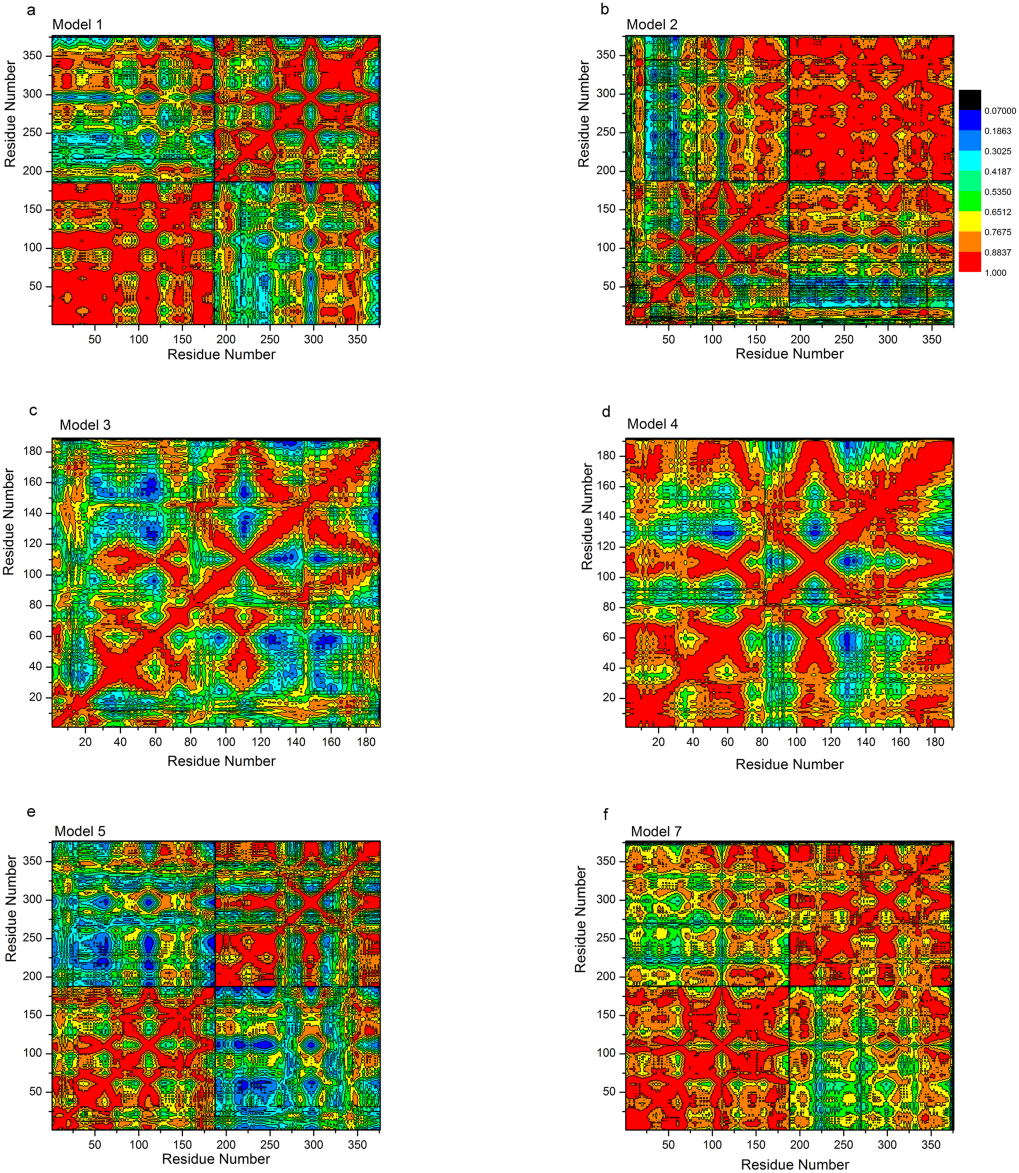
Correlation Factors analysis in Models 1, 2, 3, 4, 5 and 7. Color legends are indicated in Fig 12b.

Allosteric process results in the surface charge variation. In the close state, because of the tight “mouth” length and smaller accessible area, negative charge in loop-4 is slightly enriched than that in the open state, while most positive charge in the active site is more spread out than in the open state. To explore the allosteric communication caused by surface charges in the two states, the motion correlations of carbon/oxygen/nitrogen atoms were analyzed and the cross-correlation map constructed from the trajectory is given in Fig 12. The results show that the motion correlations between the residues ranged from high anti-correlation (black) to high correlation (red). Large correlated motions are detected in only one molecule of YhdE dimer in Model 1 and Model 2, or among residues in loop-4, loop-6 and loop-8 in Models 3 to 8, which is consistent with the result of the hydrogen bonding and RMSF analyses.

Correlation factor analysis suggests that there are large correlated motions of β4, β5 and β6 (residue number 150–200 and residue number 300–350 in Fig 12). These correlated motions reflect allosteric communications among those β-strands. It indicates that the interactions between β4, β5 and β6, together with the hydrogen bonding and electrostatic interactions, play critical roles in the allosteric communications in the catalytic domain. It shows that the motions of β5 significantly correlate with that of the loop-6 where E33 is located, which indicates the allosteric communication between β5 and loop-6. Furthermore, the closed state shows less correlation than the open state between β3 and β6, suggesting that the closed state contains a β-strand network for its binding pocket enlargement which is consistent with our pocket accessibility analysis result.

To further analyze cooperative effect, we also performed correlation factor study using the closed state of YhdE dimer with one dTTP bound. In this model, one of the active sites is filled with one dTTP molecule from the docking results. Results suggest that multiple hydrogen bonds between two β6 strands in two YhdE molecules stabilize the entire β-sheet of β3-β4-β5-β6, which acts as the foundation of binding pocket and controls its accessible area/volume. It also shows that correlation between β6 in two YhdE molecules is reduced with the dTTP binding in Model 5 but not in Model 7, indicating that dTTP could regulate allosteric communications in the YhdE open state dimer, but not in the closed state dimer. The pocket analysis suggests that in the apo-form, the active site is closer than that in dTTP-bound side. Length between Cα of A5 and Cα of E140 in apo-form side is larger than that in dTTP-bound side. This indicates that YhdE shows cooperative effect in dTTP catalytic reaction with a conformational change. Hydrogen bonding analysis suggests that strength of intermolecular Hydrogen bond is as large as those among β-strand network, indicating that the cooperative effect involves a possible transmission pathway leading to conformational changes through the β-strand network.

## Discussion

### YhdE closed state is a naturally occurred state in the absence of dTTP with high protein concentration

Our crystallization study shows that the closed conformation of YhdE is a naturally occurred state in the absence of dTTP binding. This result suggests that the closed state itself has intrinsic stability. Molecular dynamic simulations indicate that the catalytic loop in the close state is as tight as that in the open state based on the analysis of RMSD. As the “mouth” of the closed state is smaller than that in the open state and the closed state is discovered from decreasing the protein concentration, it is possible that molecular packing plays an important role in the formation of YhdE closed state. YhdE exists as dimer in solution, offering the opportunity for multi-molecular interactions. These interactions may induce conformational change of YhdE from the open to the closed state, or *vice versa*.

### YhdE open state is a substrate binding favorable conformation and its closed state is a reaction favorable conformation

**Fig 13:**
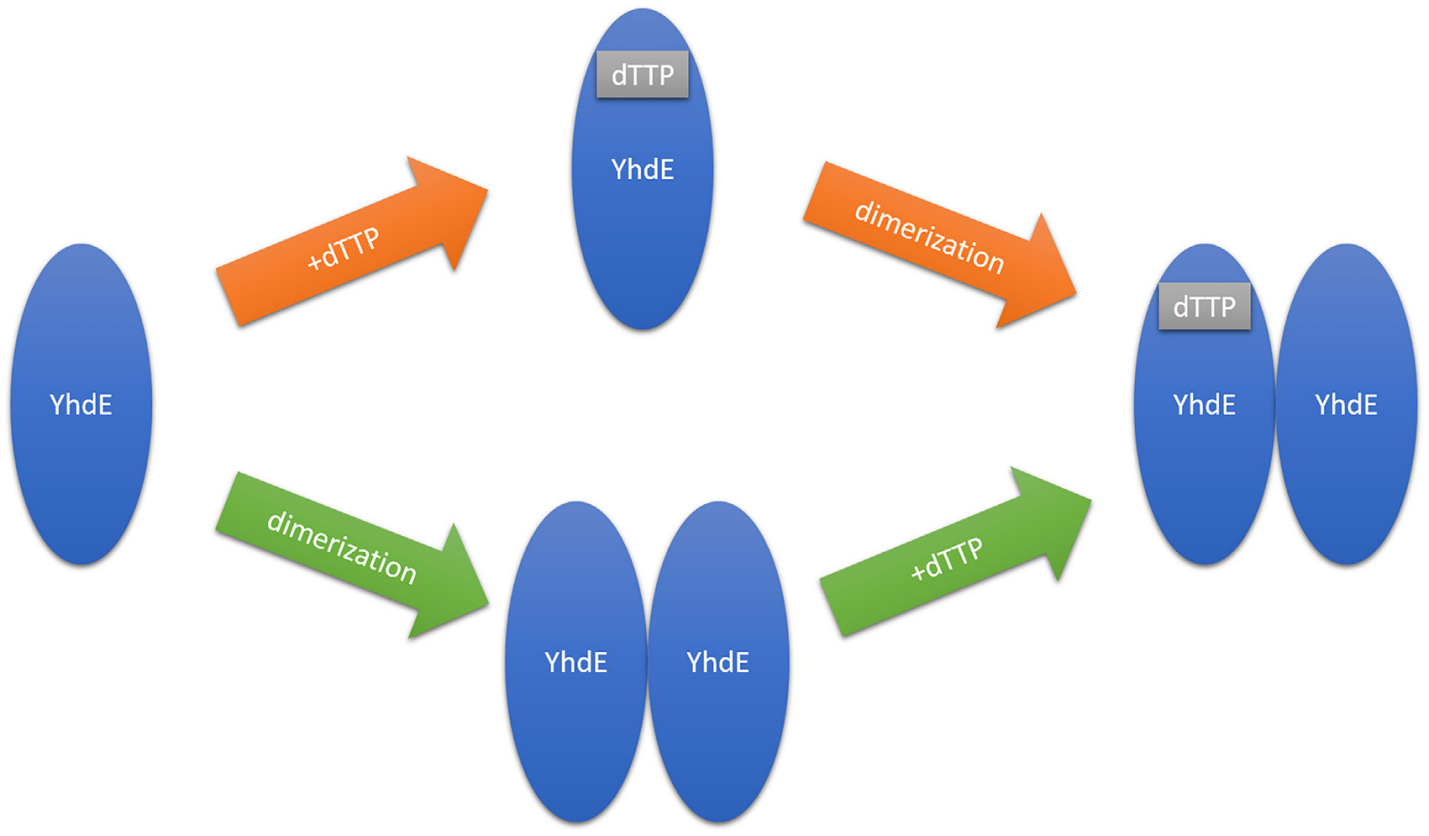
Two possible scenarios of cooperative effect in YhdE catalyzed dTTP hydrolysis. Orange arrow indicates that dTTP binds YhdE first and green arrow indicates that YhdE dimer forms first.

Crystal structures of YhdE in the open and closed states raise questions whether this conformational variation is related to dTTP binding (Fig 13). Our studies indicate that the closed state is favorable for reaction to occur, and provide evidences that the open state is more favorable for dTTP binding than the closed state. Previous studies [8] have suggested two possible explanations for dTTP binding to the YhdE open/closed states: one is that the substrate binding induces YhdE conformational change from the open to closed state, while the other one is that YhdE open/close states in complex with dTTP or other substrates are naturally occurring and the one with YhdE closed state is primed for reaction. Our calculation result is consistent with the former suggestion. The naturally existing YhdE closed state crystal predicts that “squeezing” effect determines which conformation YhdE belongs to. The average YhdE dimer conformation indicates that the closed state is a high-tension conformation which would squeeze dTTP at room temperature and may be more favorable for dTTP hydrolysis than the open state, yet binding pocket is tighter in the ‘mouth’ part and looser in the inner part in comparison with the open state. These structural variations show that the base group of the substrate may play a role in substrate-enzyme interactions and influence pocket size at the same time, while phosphate groups does not directly affect the active site.

### YhdE dimerization benefits dTTP binding, which originates from allosteric communication

As mentioned above, two β6 strands from two YhdE monomers interact to form an extended intermolecular β-sheet, which stabilizes YhdE dimer. Surface electrostatic analyses of the dimer suggest that β6 makes this intermolecular β-sheet more positively charged. This positively charged surface could favor the substrate binding, which benefits dTTP/UTP hydrolysis reaction in the apo molecule of YhdE dimer. Moreover, this intermolecular β-strand may benefit the ‘squeezing effect’ in the apo molecule when another monomer is filled with substrate. These interactions would extend existing β-strand and allosteric regulation, which accelerates dTTP/UTP hydrolysis reactions.

Correlation analysis and RMSF analysis indicate that in apo-form YhdE dimer (Model 1 and Model 2), one YhdE molecule is more flexible and other one is more rigid than compared to monomer alone. However, binding of dTTP reduces this difference, making another dTTP binding more favorable. It shows that YhdE dimerization makes allosteric regulation possible for the second dTTP binding to the active site, which is reflected in the cooperative effect.

### Catalytic reaction occurs by changing free energy decomposition among those models

Free energy studies suggest that dTTP binds to YhdE open/closed conformation with different energy decompositions in dTTP binding to YhdE dimer. YhdE conformational variations between the open state and the closed state results in K50 being less important and K80 being more important. It suggests that loop-6 becomes more important in dTTP binding to the YhdE closed state than to the YhdE open state. This reflects the conformational change of loop-6 may cause an increase of free energy of the entire complex. The whole system may release the extra free energy through substrate dTTP hydrolysis.

## Conclusions

A series of molecular dynamics simulations and free energy calculations were carried out, demonstrating that it is energetically more favorable for YhdE dimerization being as the first reaction step or the dimerization-first model is favorable than the binding-first model, which further confirms that the cooperative effect plays an important role in the binding of the first dTTP. The energy decomposition predicts that the contribution of the free-energy contributing to the cooperative process comes mainly from the key residues R10, R11, K50 and K80 in YhdE. It has been observed that dTTP binding to the closed state of YhdE causes gradual size decrease of the cleft between V28 and S36 in loop-4 with the movement of loop-6. Such changes result in binding pocket variation between YhdE open state and YhdE closed state, suggesting that the binding pocket in the close state is more “closed” because outer residues are extended away from the pockets, which contributes little to the formation of pocket.
